# Increased expression of oxyproteins in the optic nerve head of an in vivo model of optic nerve ischemia

**DOI:** 10.1186/1471-2415-12-63

**Published:** 2012-12-05

**Authors:** Joon Mo Kim, Yu Jeong Kim, Dong Myung Kim

**Affiliations:** 1 Department of Ophthalmology, Kangbuk Samsung Hospital, Sungkyunkwan University School of Medicine, Seoul, Korea; 2 Seoul National University Hospital Clinical Research Institute, Seoul, Korea; 3 Department of Ophthalmology, Seoul National University College of Medicine, 28 Yongon-Dong, Chongno-Gu, Seoul, 110-744, Korea

**Keywords:** Ischemia, Optic nerve, Oxyproteins, Oxyblot

## Abstract

**Background:**

To investigate the effects of microvascular compromise on the expression of oxidative proteins in the optic nerve head.

**Methods:**

Endothelin-1 (0.1 μg/day) was delivered to the perineural region of the anterior optic nerve by osmotically driven minipumps for two, four, and eight weeks in ten rabbits, respectively. As a control, a balanced salt solution was delivered for two and eight weeks in five rabbits, respectively. Expression of oxyproteins in the cornea, vitreous, retina, and optic nerve head for each time period was determined using the OxyBlot protein oxidation detection kit. Retina was stained with H&E and TUNEL for histological examination.

**Results:**

There was a significant increase in the expression of oxyproteins in the optic nerve head after two weeks of endothelin-1 administration (p < 0.001, Mann Whitney U test). In contrast, there was no expression of oxyproteins in the cornea, retina, or vitreous. The number of cells in the retinal ganglion cell layer, inner nuclear layer, and outer nuclear layer decreased remarkably with time in the endothelin-1-treated group. Furthermore, the inner and outer nuclear layers, as well as the inner and outer plexiform layers, became thinner over time.

**Conclusions:**

Administration of endothelin-1 to the microvasculature of the optic nerve leads to increased expression of oxyproteins in the optic nerve head and loss of retinal ganglion cells.

## Background

Glaucoma, a leading cause of blindness, is characterized by progressive retinal ganglion cell loss and excavation of the optic nerve head. However, the pathophysiologic mechanisms leading to glaucomatous damage have not yet been fully elucidated. Elevated intraocular pressure is considered to be the primary cause of glaucomatous damage. In addition, clinical studies have suggested that microcirculatory changes may have a role in glaucoma, either as the primary abnormality or as a cofactor that increases susceptibility to pressure damage [1-6]. Furthermore, the relevance of oxidative stress to the pathogenesis of glaucoma has been demonstrated in cell and animal studies [7,8]. Elevated pressure causes oxidative stress in the extracellular matrix of the trabecular meshwork, which in turn increases intraocular pressure and leads to apoptosis of retinal ganglion cells [9-11].

Arterial sclerosis and increased levels of ET-1 can decrease blood flow to the optic nerve and trigger oxidative damage. ET-1, a strong vasoconstrictor, increases vascular resistance, reduces blood flow to the eye, and induces apoptosis of retinal ganglion cells. ET-1 has also been shown to bind ET_A_ and ET_B_ receptors in the optic nerves of rabbits and humans [12,13]. Furthermore, both glaucoma patients and genetically-modified rats have been shown to have high intraocular concentrations of ET-1 [14,15]. Blood flow to the optic nerve is unstably decreased, and minimal reperfusion is observed after exposure to ET-1 [16]. It is well-known that repeated chronic oxidative stress caused by reperfusion leads to the loss of the retinal ganglion cells [17]. Furthermore, a previous study found that ET-1 is increased and metalloproteinases are upregulated in glaucoma patients, [18] suggesting that ischemia and oxidative stress are important factors related to glaucomatous optic nerve damage.

Increased levels of ET-1 have been known to lead to a reduction in blood flow in both the choroid and the optic nerve head [19]. ET-1 constricts vessels both directly and indirectly by increasing the sensitivity to other vasoconstrictive hormones such as norepinephrine, 5-hydroxytryptamine, and angiotensin-II. An increase in circulating ET-1 markedly reduces blood flow in the eye [20]. If the concentration of ET-1 is even higher, it causes vasospasm [21]. The stimulation of ET receptors on smooth muscle cells or pericytes increases cytoplasmic calcium, both by influx into the cell, as well as by liberation of calcium from the internal storage [22]. High levels of ET-1 in the eye cause pro-inflammatory cytokine overproduction and oxidative stress pathway activation, as well as reduced trophic support and oxygen delivery to the retina [23].

The purpose of this study was to investigate whether increased expression of oxyproteins in the optic nerve head is associated with microcirculatory compromise of the optic nerve. To this end, we used rabbits in which endothelin-1 (ET-1; Peptides International, Louisville, KY) was delivered to the perineural region of the anterior optic nerve by an osmotically-driven minipump (Alzet Minipumps; Alza Corporation, Palo Alto, CA) as a model for optic nerve ischemia [24,25]. A reduction of approximately 38% of the optic nerve blood flow in ET-1-administered eyes compared with control eyes was previously demonstrated in this model [26].

## Methods

Forty male New Zealand white rabbits weighing 2.5 to 3.5 kg were used for this study. All experiments conformed to the Association for Research in Vision and Ophthalmology statement for the Use of Animals in Ophthalmic and Vision Research. Rabbits were anesthetized with zoletin and xylazine, after which anterior optic nerve ischemia was induced as previously described by Cioffi *et al*. and Kim *et al*. [24,25,27,28]. Briefly, ET-1 was delivered to the perineural region of the anterior optic nerve by osmotically driven minipumps at a controlled and constant flow rate (0.5 μL/h). Minipumps were implanted in a surgically-created space superior and nasal to the right eyes. A polyethylene delivery tube was directed from the minipump through the upper eyelid into a surgically-created superior-temporal sub-tenon channel under the superior rectus muscle. It was fixed in place using a scleral fixation suture adjacent to the optic nerve and its vascular supply. A 0.1 μg/day dosage of ET-1, which was diluted with balanced salt solution (BSS) was delivered for two (Group II), four (Group III), and eight weeks (Group IV) in ten rabbits, respectively. As a control, BSS was delivered for two (Group Ia) and eight weeks (Group Ib) in five rabbits, respectively.

Rabbits were sacrificed and enucleated after two, four, and eight weeks of ET-1 administration and after two and eight weeks of BSS administration. After enucleation, oxidative proteins in the cornea, vitreous, retina, and optic nerve head were measured for each time period using the OxyBlot protein oxidation detection kit (Chemicon, Billerica, MA, USA) in triplicate. Protein oxidation occurs as a result of generation of reactive oxidative products through either direct or indirect pathways. The most common in vivo oxidative reaction is the formation of reactive products with carbonyl functional groups. The detection kit utilizes an immunoblot that quantifies the level of oxidized proteins formed by reactions with carbonyls. Briefly, samples were dissolved in radioimmunoprecipitation assay (RIPA) buffer supplemented with 50 mM dithiothreitol to protect against further oxidation. The refined protein samples were then analyzed using the OxyBlot protein oxidation detection kit. Using this kit, carbonyl groups were converted to 2,4-dinitrophenylhydrazone (DNPH) by reacting with 2,4-dinitrophenylhydrazine. The resulting dinitrophenyl-derivatized protein samples were blotted onto a membrane filter, incubated with a peroxidase-antibody conjugate that binds to the dinitrophenyl section of the protein and goat anti-rabbit IgG, and visualized with chemiluminescent reagents. Protein expression was quantified by densitometry. To compare the results of OxyBlot protein oxidation detection, relative densitometric values of the treated groups were compared to the control groups. In addition, retinas from Groups II, III, IV, and Ib were subjected to H&E and TUNEL staining for histological examination. TUNEL staining was performed using an ApopTag Peroxidase In Situ Apoptosis Detection Kit (Millipore, USA). The sections were treated with proteinase K (DAKO, USA) and blocked by hydrogen peroxide. Following the washes, the sections were incubated with an enzyme buffer containing terminal deoxynucleotidyl transferase and dUTP. The apoptotic cells were detected by DAB substrate chromogen system (DAKO).

## Results

**Figure 1:**
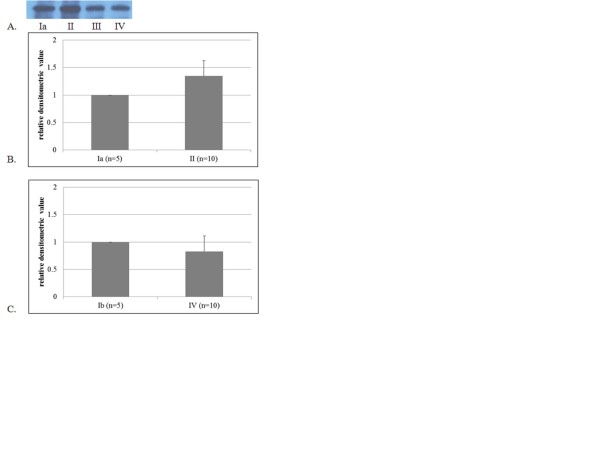
**Results of OxyBlot protein detection (A) and relative densitometric values of oxyprotein expression in the optic nerve head (B, C).** Blotting was semi-quantified using densitometry. To compare the results of OxyBlot protein oxidation detection, relative densitometric values of treated groups were compared to control groups. Endothelin-1 (0.1 μg/day) was delivered to the perineural region of the anterior optic nerve by osmotically-driven minipumps for two (II), four (III), and eight weeks (IV). As a control, balanced salt solution was delivered for two (Ia) and eight weeks (Ib). There was a significant increase in the expression of oxyprotein after two weeks of endothelin-1 administration (p < 0.001, Mann Whitney U test). However, there was no difference between the two groups at eight weeks.

**Figure 2:**
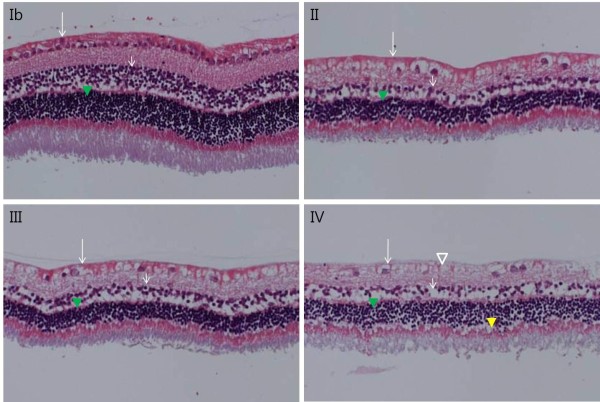
**Retina histological findings (H&E staining, x 200).** An endothelin-1 dosage of 0.1 μg/day was delivered for two (II), four (III), and eight weeks (IV), and balanced salt solution was delivered for eight weeks (Ib) to the perineural region of the anterior optic nerve. Compared to Group Ib, the number of cells in the retinal ganglion cell layer (long arrow), the inner nuclear layers (short arrow), and the outer nuclear layer (green arrowhead) were remarkably decreased in Groups II, III, and IV. Vacuoles without a nucleus (empty arrowhead) were found in endothelin-1-treated groups. In addition, thinning of the inner and outer nuclear layers and the inner and outer plexiform layers was noted. The outer limiting membrane, composed of the zonula adherens, exhibited loosening and widening (yellow arrowhead).

**Figure 3:**
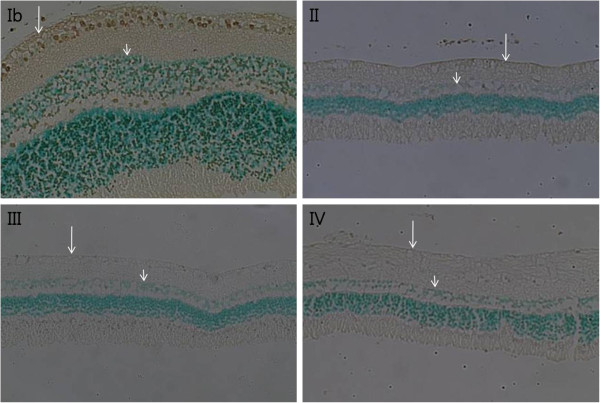
**Retina histological findings (TUNEL staining, x 200).** An endothelin-1 dosage of 0.1 μg/day was delivered for two (II), four (III), and eight weeks (IV) and balanced salt solution was delivered for eight weeks (Ib) to the perineural region of the anterior optic nerve. Apoptotic cells (gray) were observed mainly in the ganglion cell layer (long arrow) and inner nuclear layer (short arrow) in Group Ib. However, very few apoptotic cells were observed in Groups II, III, and IV.

Oxyprotein expression was significantly increased in the optic nerve head in Group II compared with Group Ia (p < 0.001, Mann Whitney U test, Figure 1). However, after two weeks, expression had decreased. Furthermore, there was no difference in the expression of oxyproteins between Groups IV and Ib. In contrast, oxidative proteins were not expressed in the cornea, vitreous, or retina (data not shown). The intercellular structures in each retinal cell layer were destroyed, the intercellular space loosened, and the cell number in the cell layer appeared to have decreased. The number of cells in the retinal ganglion cell layer, inner nuclear layer, and outer nuclear layer decreased notably with time in groups treated with ET-1 (Figure 2). In addition, the inner and outer nuclear layers, as well as the inner and outer plexiform layers, thinned over time. In Group IV, the outer limiting membrane exhibited both loosening and widening. On TUNEL staining, very few apoptotic cells were observed in the ET-1-treated groups, whereas none were observed in the control groups (Figure 3).

## Discussion

Oxidative damage is the result of oxidative insults due to the production of reactive oxygen species and reactive nitrogen species generated by local ischemia. Accordingly, such increases in oxidative protein damage can affect cellular integrity [29]. In the present study, we evaluated whether the expression of oxyproteins was increased in the optic nerve head after ET-1-induced optic nerve ischemia. The number of cells in the retinal ganglion cell layer and inner and outer nuclear layers, as well as the thicknesses of the cell layers, decreased over time. The thickness of the inner and outer plexiform layer was also decreased. These results suggest that ischemia-induced oxidative stress can damage both the retina and optic nerve.

The term ‘oxidative stress’ is used to indicate excessive increases in the levels of reactive oxygen and nitrogenous compounds compared with normal physiological intracellular levels [30]. The eye in particular is exposed to light, radiation, oxygen, and chemicals. In addition, noxious reactive oxygen/oxygen-derived free radicals are produced normally during cellular metabolism of oxygen through processes such as electron hopping and oxidation-reduction [14,15,31]. As such, various antioxidative defense mechanisms have evolved to protect cells from oxidative damage [31,32]. The oxides produced via physiological metabolism are eliminated by antioxidants, and damaged DNA and proteins are repaired. However, some structural abnormalities resulting from excessive oxidative stress may be beyond the capacity of physiologic recovery mechanisms [33].

Excessive free radicals in a cell can disrupt intracellular structures, including nucleic acids, lipids, and proteins [34,35]. Furthermore, proteins are the primary target of oxygen-derived free radicals [36-39]. An excess in reactive oxygen species induced by oxidative stress directly triggers insults in the retina, including mitochondrial dysfunction, [40] induction of apoptosis, increased neurotoxin production, weakening of the neuroprotective functions of glial cells, and activation of immune-mediated neuronal injuries. Indeed, oxidative stress in retinal ganglion cells and retinal proteins may be the cause of optic disc deformities [10,11].

Quantification of intracellular oxyproteins is necessary for a comprehensive understanding of oxidative stress. Measurement of DNPH protein bound carbonyl derivatives is one of the most commonly used strategies to quantify oxyproteins, and DNPH is quite sensitive with respect to immunodetection using specific antibodies [41]. In our study, following two weeks of treatment with ET-1, the expression of oxyproteins was significantly increased compared to the control group. However, at four and eight weeks, the level of oxyprotein expression was equal to or slightly decreased compared to the control group. When a protein undergoes irreversible oxidative modifications and cannot be repaired, it is eliminated through degradation. The mechanisms of protein elimination are through either proteome-mediated proteolysis [42] or chaperone-mediated autophagy [43]. In either case, vulnerable cells damaged by ischemia and oxidative stress may have their damaged proteins eliminated by these mechanisms, thereby allowing resistant cells to persist. Thus, equivalent levels of oxyprotein expression at four and eight weeks suggests the presence of a relative increase in stressful environments as well as expression of oxidative proteins in the ET-1- administered group compared to the control group.

The results of the present study also demonstrate the presence of oxidative stress in the control group. Specifically, apoptosis was more apparent in the control group than in the ET-1-treated group, which may be a reflection of the effects of natural stress such as light, chemicals, or radiation. Furthermore, the basal production of reactive oxygen species, which are regulated by antioxidants, is part of normal cellular redox homeostasis. Thus, the balance between native and oxidatively-damaged proteins depends on the rates of protein biosynthesis, oxidative modification, and oxidized protein elimination [44].

In our results, there was no expression of oxyproteins in the retina by oxyblot, yet cell loss was observed in retinal layers. In this study, we analyzed separation of the optic nerve head from the retinal layer. For this analysis, we collected optic nerve head tissue, which protrudes into the globe. The optic nerve head consists mainly of the surface nerve fiber layer and the prelaminar layer. Those layers include mainly axons and some deep retinal layers. The structure of the retina containing whole layer is different from that of the surface retinal layers. Therefore, if oxyprotein is expressed more in the axons, the oxyblot result will be different from that of the thin axons of the existing retinal layers. Since oxyblot is a quantitative method, a certain amount of oxyprotein is needed for detection. Therefore, the absence of detection can be attributed to a low amount of oxyprotein in the tissue. To date, there have been no studies regarding whether oxyprotein can migrate along the axon. In a previous animal model study, the injection of ET-1 into the posterior chamber caused a significant constriction of retinal vessels and impaired retrograde axonal transport by decreasing the number of retinal ganglion cells [45]. The number of retinal ganglion cells decreased by 44.2% four weeks after the injection of ET-1 into the posterior chamber. For this reason, apoptosis in retinal ganglion cells should be rarely observed in the ET-1-treated group.

## Conclusion

The exact mechanisms that lead to damage of the optic nerve head and retinal ganglion cells in glaucoma are still unknown, especially in glaucoma without high intraocular pressure. However, multiple mechanisms and factors are undoubtedly involved in glaucoma. The model used in the present study simulates glaucoma caused by microcirculatory compromise in terms of optic nerve blood flow reduction. Thus, the current demonstration of increased expression of oxyprotein in the optic nerve head in optic nerve ischemia justifies its use in studies aimed at both understanding and inhibiting the mechanisms associated with retinal ganglion cell damage.

## Competing interests

The authors declare that they have no conflict of interest.

## Authors’ contributions

Literature screening and selection was performed by JMK and DMK. JMK, YJK, and DMK participated in the design of the study, and JMK performed the statistical analysis. Preparation of the first draft of the manuscript was done by JMK, and review and approval of the manuscript was performed by YJK and DMK. All authors read and approved the final manuscript.

## Financial disclosure

The authors have no proprietary or commercial interest in any of the materials mentioned in this article.

## Pre-publication history

The pre-publication history for this paper can be accessed here:

http://www.biomedcentral.com/1471-2415/12/63/prepub
